# Fluorescent Fusion Proteins of Soluble Guanylyl Cyclase Indicate Proximity of the Heme Nitric Oxide Domain and Catalytic Domain

**DOI:** 10.1371/journal.pone.0011617

**Published:** 2010-07-15

**Authors:** Tobias Haase, Nadine Haase, Jan Robert Kraehling, Soenke Behrends

**Affiliations:** Institut für Pharmakologie, Toxikologie und Klinische Pharmazie, Technische Universität Braunschweig, Braunschweig, Germany; University of Illinois at Chicago, United States of America

## Abstract

**Background:**

To examine the structural organisation of heterodimeric soluble guanylyl cyclase (sGC) Förster resonance energy transfer (FRET) was measured between fluorescent proteins fused to the amino- and carboxy-terminal ends of the sGC β_1_ and α subunits.

**Methodology/Principal Findings:**

Cyan fluorescent protein (CFP) was used as FRET donor and yellow fluorescent protein (YFP) as FRET acceptor. After generation of recombinant baculovirus, fluorescent-tagged sGC subunits were co-expressed in Sf9 cells. Fluorescent variants of sGC were analyzed *in vitro* in cytosolic fractions by sensitized emission FRET. Co-expression of the amino-terminally tagged α subunits with the carboxy-terminally tagged β_1_ subunit resulted in an enzyme complex that showed a FRET efficiency of 10% similar to fluorescent proteins separated by a helix of only 48 amino acids. Because these findings indicated that the amino-terminus of the α subunits is close to the carboxy-terminus of the β_1_ subunit we constructed fusion proteins where both subunits are connected by a fluorescent protein. The resulting constructs were not only fluorescent, they also showed preserved enzyme activity and regulation by NO.

**Conclusions/Significance:**

Based on the ability of an amino-terminal fragment of the β_1_ subunit to inhibit activity of an heterodimer consisting only of the catalytic domains (α_cat_β_cat_), Winger and Marletta (*Biochemistry* 2005, 44:4083–90) have proposed a direct interaction of the amino-terminal region of β_1_ with the catalytic domains. In support of such a concept of “trans” regulation of sGC activity by the H-NOX domains our results indicate that the domains within sGC are organized in a way that allows for direct interaction of the amino-terminal regulatory domains with the carboxy-terminal catalytic region. In addition, we constructed “fluorescent-conjoined” sGC's by fusion of the α amino-terminus to the β_1_ carboxy-terminus leading to a monomeric, fluorescent and functional enzyme complex. To our knowledge this represents the first example where a fluorescent protein links two different subunits of a higher ordered complex to yield a stoichometrically fixed functionally active monomer.

## Introduction

Guanylyl cyclase (EC 4.6.1.2) catalyzes the reaction of GTP to the second messenger molecule cGMP. The pharmacologically important membrane spanning forms act as cell surface receptors for extracellular peptides e.g. atrial natriuretic peptide (ANP) [1]. Soluble guanylyl cyclase (sGC) is a time-tested target for drugs like glyceryl trinitrate that release nitric oxide (NO) (for review see [2]). Innovative drugs are now being developed clinically that either sensitize the enzyme for activation by NO or activate the enzyme independently of NO [3], [4]. The enzyme forms relevant for treatment of coronary artery disease, (pulmonary) hypertension, erectile dysfunction or arterial occlusive disease consist of a β_1_ subunit and an α subunit (either α_1_ or α_2_). The most prevalent heterodimeric form α_1_/β_1_ has been purified from native tissue as a heme containing enzyme and has been characterized extensively by several groups. Despite enormous interest in the structure of the enzyme in academia and industry, the heterodimeric holoenzyme has so far resisted all crystallization attempts.

Current information on the structure of the enzyme comes from homology models of proteins with a known structure that are related to functional domains within sGC. The carboxy-terminal parts of the α_1_ and β_1_ subunits are homologous to the C1 and C2 domains of mammalian adenylyl cyclase and a eukaryotic guanylyl cyclase from *Chlamydomonas reinhardtii* [5]. The amino-terminal region is homologous to bacterial O_2_ sensor proteins and is therefore referred to as heme nitric oxide oxygen (H-NOX) binding domain [6], [7]. Homology models for sGC-β_1_ H-NOX [6], [8] based on these bacterial proteins suggest that it is central for full activation by NO [9]. The H-NOX *associated* domain (H-NOXA) is a central subdomain flanked by the amino-terminal H-NOX domain and a carboxy-terminal predicted coiled coil [10], [11]. H-NOXA domains are found in both sGCα and sGCβ_1_ and a number of bacterial homologues [12]. Based on the crystal structure of the dimerized domain of a signal transduction histidine kinase from *Nostoc punctiforme* it has been shown to have a Per-Arnt-Sim (PAS) fold [13]. For better distinction from H-NOX we will refer to the H-NOXA domain as the PAS-domain in the current paper. The PAS domain seems to be involved in heterodimerization of sGC [13]. Very recently the central coiled coil region of the sGC β_1_ subunit has been crystallized as a homodimer and revealed an anti-parallel arrangement of the monomers [14]. Nevertheless, based on homology modeling the authors suggest a parallel arrangement of the coiled coil region in heterodimeric enzymes as in the membrane bound guanylyl cyclase forms.

There are at least two alternatives how NO binding to the amino-terminal β_1_ H-NOX may influence the conformation of the carboxy-terminal catalytic domain. The classic idea has been that NO binding to β_1_ H-NOX induces a conformational shift of the whole enzyme that is transmitted to the carboxy-terminal catalytic domain through the PAS and coiled coil domains. This would be analogous to membrane bound guanylyl cyclase A where binding of ANP to the extracellular domain is clearly separated from the intracellular catalytic domain by the plasma membrane. A more novel concept is that β_1_ H-NOX may directly interact with the catalytic region: Winger and Marletta have expressed and purified the heterodimeric catalytic domain in *E. coli* [15]. They could show that the presence of increasing amounts of the separately expressed, isolated β_1_ H-NOX domain led to concentration dependent inhibition of catalytic activity. Although NO had no effect on the inhibition by β_1_ H-NOX they suggested a model where the H-NOX domain of the β_1_ subunit interacts directly with the catalytic domain [15]. A prerequisite of this model would be close proximity of the H-NOX subdomain and the catalytic region in the intact heterodimeric enzyme. Although structural homology models are available for subdomains, an analysis of their relative position to each other is lacking.

In the current paper we use FRET analysis of fluorescent fusion proteins to show close proximity of the amino-terminal H-NOX domains and the catalytic region supporting the model by Winger and Marletta. In addition, we tested this structural organization by fusion of the carboxy-terminus of the β_1_ subunit to the amino-terminus of the α_1_ subunit. In accordance with the prediction this monomeric fusion of the formerly heterodimeric NO-receptor results in a functional NO-sensitive enzyme. This confirms the concept of close proximity of H-NOX domains and the catalytic domain. Our data thus make it likely that significant differences exist within the guanylyl cyclase family with respect to the communication of the regulatory molecules NO and ANP from the amino-terminus to the carboxy-terminus of guanylyl cyclases.

## Results

### Expression and functional characterization of fluorescent tagged sGC subunits

**Figure 1:**
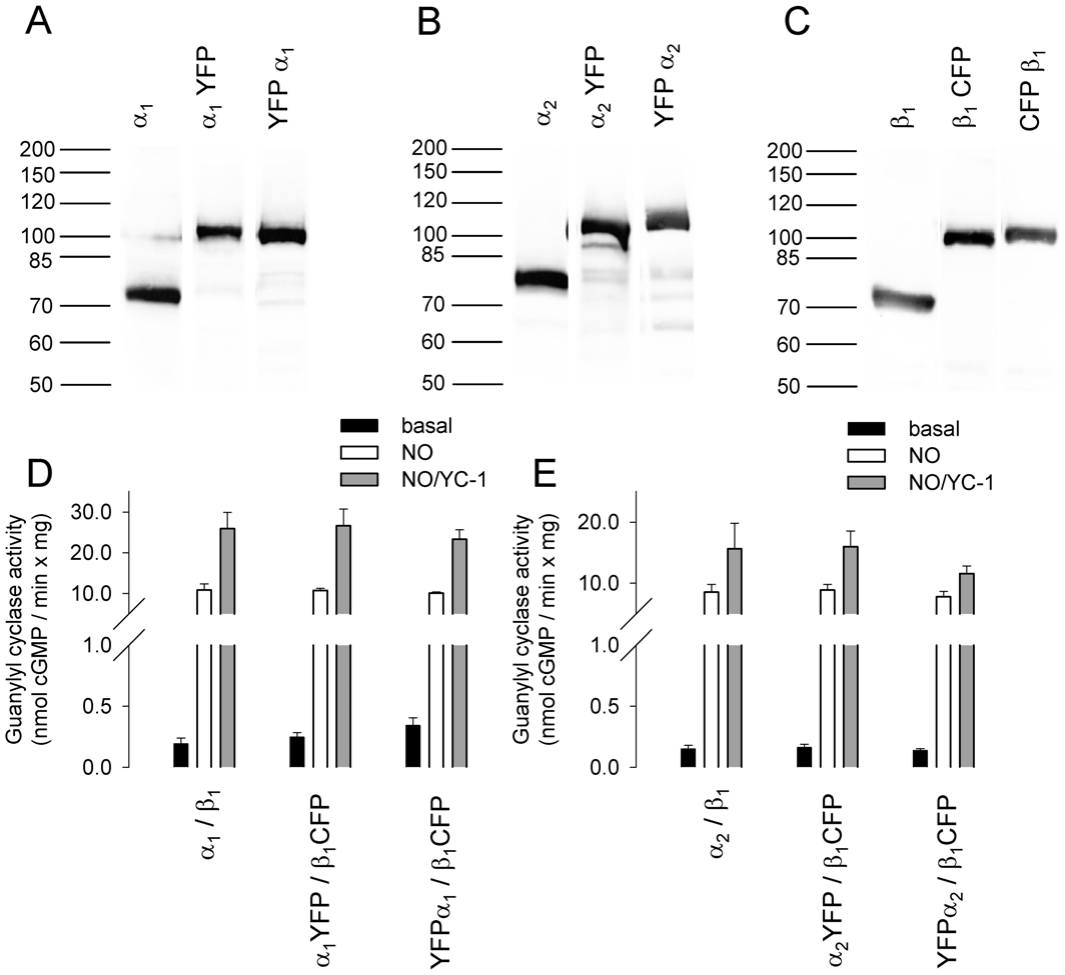
Analysis of fluorescent tagged heterodimeric sGC subunits in cytosol of infected Sf9 cells. Western blot analysis with antibodies against the α_1_ subunit (**A**) α_2_ subunit (**B**) and β_1_ subunit (**C**). Specific sGC activity of the α_1_/β_1_ heterodimer (**D**) and the α_2_/β_1_ heterodimer (**E**) in comparison with the respective fluorescent tagged heterodimeric sGC. The lanes shown for the respective antibody are assembled from the same Western blot. Data are mean ± S.E. of three independent experiments performed in duplicate.

A series of fluorescent tagged sGC subunits were constructed (Supplementary Methods S1) and expressed in Sf9 cells together with the dimerizing partner subunit. On Western immunoblots using antibodies directed against the β_1_, α_1_, and α_2_ subunit, fluorescent-tagged sGC subunits were detected at the expected molecular masses predicted from their deduced amino acid sequence (Fig. 1 A–C). By titrating the multiplicity of infection (MOI) of donor and acceptor tagged sGC subunit baculoviruses according to Western immunoblot results in coinfected cytosolic fractions, expression levels of donor and acceptor tagged subunits were adjusted to equal levels. Expression levels of wild type sGC and fluorescent tagged sGC subunits were also similar (see Fig. 1 A–C). Spectrofluorometric studies showed that the fusion of CFP or YFP to the sGC subunits did not alter the spectral properties of the fluorescent proteins (Supplementary Fig. S1).

**Figure 2:**
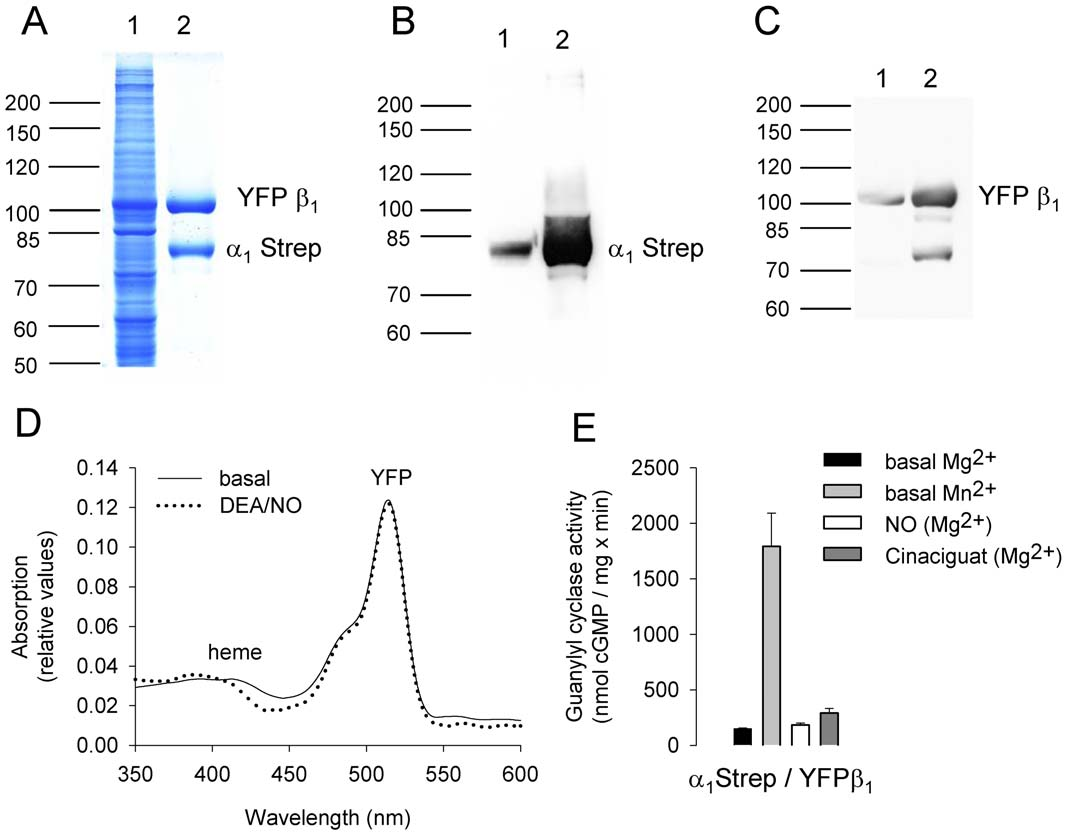
Analysis of purified heterodimeric sGC with an amino-terminally tagged β_1_ subunit. Coomassie blue staining (**A**) and Western blot analysis with antibodies directed against the α_1_ subunit (**B**) and the β_1_ subunit (**C**) using cytosolic fraction (lane 1) or purified sGC (lane 2). Absorption spectra of purified sGC under basal conditions (solid line) and in the presence of 100 µM DEA/NO (dotted line) (**D**). Specific sGC activity of purified sGC assayed with Mg^2+^ (black column) and Mn^2+^ (grey column) as cofactor and in the presence of 100 µM DEA/NO (white column) and 10 µM Cinaciguat (dark grey column) (**E**).

To ensure that attaching fluorescent proteins to sGC subunits would not affect the formation of a functional heterodimeric enzyme complex, we assayed specific guanylyl cyclase activity. Specific guanylyl cyclase activity was measured in cytosolic fractions of infected Sf9 cells under basal conditions, in the presence of the NO donor DEA/NO (100 µM) or in the presence of both the NO donor DEA/NO (100 µM) and the sGC stimulator YC-1 (100 µM). Co-expression of non-tagged sGC α_1_, and α_2_ subunits with the non-tagged sGC β_1_ subunit resulted in an activation profile characteristic of the native, heme-containing enzyme (Fig. 1 D, E). The fluorescent-tagged sGC combinations αYFP/β_1_CFP, YFPα/β_1_CFP displayed NO and NO/YC-1 stimulated sGC activity similar to the non-tagged sGC heterodimer (see Fig. 1 D, E). Co-expression of the α subunit with the amino-terminal fusion of FP's to the β_1_ subunit led to loss of NO sensitivity with preserved basal activity of the purified enzyme. Purification of the enzyme complex via Strep-Tag chromatography showed that the amino-terminally tagged β_1_ subunit is capable to dimerize with the α subunit as shown by Coomassie blue staining (Fig. 2 A–C). Spectral analysis of the fluorescent-tagged enzyme complex showed an absorption peak at 515 nm due to YFP (see Supplementary Fig. S1), but virtually no Soret band (Fig. 2 D). The apparently heme free enzyme complex showed basal enzyme activity but, no sensitivity towards NO. Surprisingly it could not be activated by the heme independent sGC modulator Cinaciguat (see Fig. 2 E). To confirm preservation of basal activity of the enzyme complex, specific sGC activity was additionally measured in the presence of Mn^2+^ instead of Mg^2+^ as cofactor as this is known to increase non NO-stimulated activity.

### FRET study of fluorescent-tagged sGC variants

**Figure 3:**
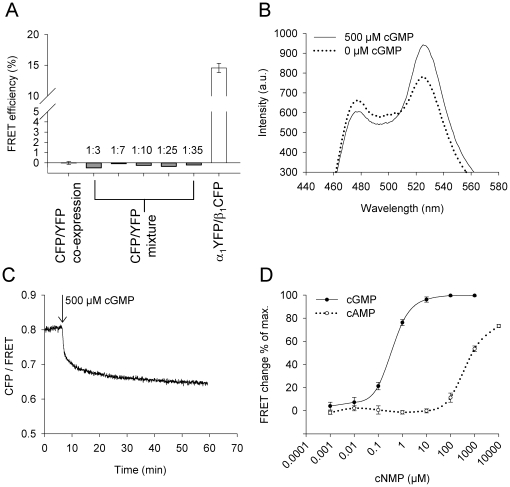
FRET analysis of positive- and negative control samples in cytosol of infected Sf9 cells. Co-expression (black column) and mixture (grey columns, CFP emission: YFP emission) of CFP and YFP yielded no FRET (**A**). Fluorescent spectra of YFP-GAFA-CFP after excitation at 436 nm, before (dotted line) and after addition of 500 µM cGMP (**B**). The ratio of intensities in the CFP channel and FRET channel decreases after addition of 500 µM cGMP, indicating an increase in FRET (**C**). Concentration-response curves of YFP-GAFA-CFP for cGMP and cAMP (n = 3) (**D**).

To examine if the fluorescent proteins tagged to the termini of sGC subunits are in proximity to each other we looked for occurrence of FRET. FRET studies were performed in cytosolic fractions of Sf9 cells infected with the respective recombinant baculovirus in a spectrofluorometer at 37°C. As a negative control for the FRET experiments the fluorescent proteins CFP and YFP were co-expressed in Sf9 cells. In the respective cytosolic fractions no FRET was detectable (Fig. 3 A). Cytosolic fractions containing singly expressed CFP, and YFP, respectively, were mixed in different ratios. In these additional negative controls no FRET was detectable (Fig. 3 A). As a FRET positive control we used a sensor protein based on the cGMP binding domain of the phosphodiesterase 5 (PDE 5 A). This fusion protein consists of the 165 amino acids GAFA domain of phosphodiesterase 5 sandwiched between CFP and YFP and is similar to cGES-DE5 [16]. After expression of the YFP-GAFA-CFP fusion protein in Sf9 cells, FRET was detected in the respective cytosolic fractions. Moreover, FRET efficiency of YFP-GAFA-CFP increased in a cGMP dependent manner. Similar to cGES-DE5 the FRET change was about 16% with EC_50_-values for cGMP and cAMP of 0.38±0.02 µM and 451.5±48.8 µM, respectively (Fig. 3 B–D). These results indicate that the measured FRET in spectrofluorometric studies reflect the specific energy transfer between CFP and YFP.

#### sGC α_1_/β_1_ isoform

**Figure 4:**
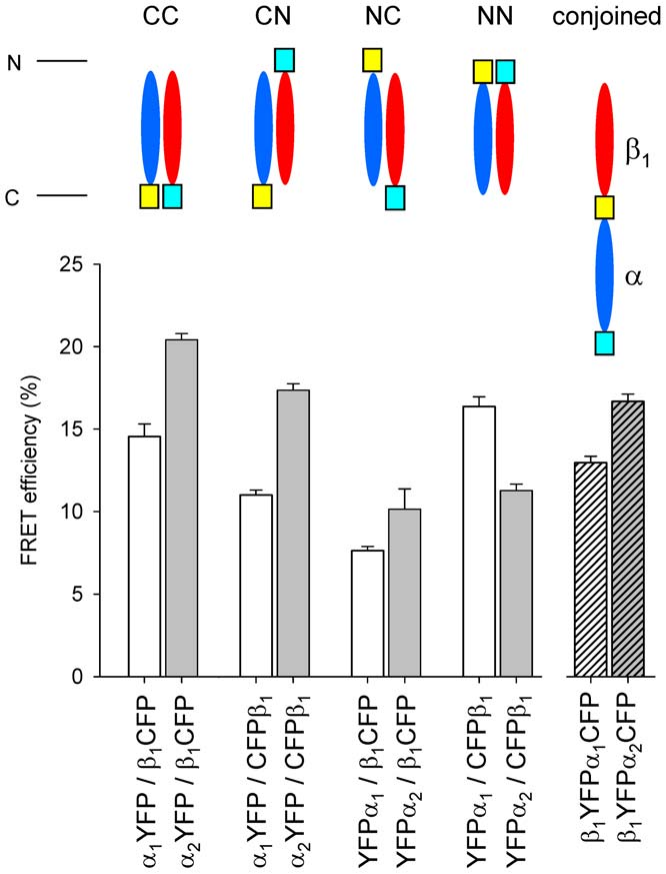
FRET analysis of heterodimeric fluorescent tagged sGC and fluorescent- conjoined sGC variants. FRET efficiencies of fluorescent tagged sGC heterodimers with the α_1_ subunit (white columns), the α_2_ subunit (grey columns) and conjoined fluorescent tagged sGC variants (hatched columns). The abbreviations given above the schematic representations indicate the terminus that is fused to the fluorescent protein. Data are mean ± S.E. of at least three independent experiments.

In the α_1_/β_1_ enzyme isoform, co-expression of carboxy-terminally (CC) tagged subunits resulted in fully active enzyme complexes that showed a FRET efficiency of 14.5% (Fig. 4). Co-expression of amino-terminally (NN) tagged subunits showed basal activity and a FRET efficiency of 16.4%. Combinations of amino-terminally and carboxy-terminally tagged subunits (CN, NC) also showed FRET, but to a significantly lower extent. The combination of carboxy-terminally tagged α_1_ subunit and amino-terminally tagged β_1_ subunit (CN) showed basal enzyme activity and a FRET efficiency of 11%. The combination of amino-terminally tagged α_1_ subunit and carboxy-terminally tagged β_1_ subunit (NC) resulted in the formation of a fully active enzyme complex that showed a FRET efficiency of 7.6%. The results from FRET measurements indicate that the carboxy- and amino-terminal domains of each subunit (CC, NN) are in closer proximity to each other than the combinations of amino- and carboxy-terminally tagged subunits (CN, NC). Nevertheless, occurrence of FRET between the combinations of amino- and carboxy-terminally tagged subunits (CN, NC) indicates that these domains are in sufficient close proximity to allow energy transfer between the fluorescent proteins (see Fig. 4, Table 1).

**Table 1:** FRET efficiencies of fluorescent-tagged sGC heterodimers and fluorescent-conjoined sGC.

		Efficiency (%)			Distance (Å)		
α_1_YFP/β_1_CFP	CC	14.5	±	0.8	65.9	±	0.7
α_2_YFP/β_1_CFP	CC	20.4	±	0.4	61.5	±	0.2
YFP-18aa-CFP *		20.0			61.7		
α_1_YFP/CFPβ_1_	CN	11.0	±	0.3	69.4	±	0.4
α_2_YFP/CFPβ_1_	CN	17.4	±	0.4	63.6	±	0.3
YFP-28aa-CFP *		13.1			67.1		
YFPα_1_/β_1_CFP	NC	7.6	±	0.3	74.3	±	0.5
YFPα_2_/β_1_CFP	NC	10.1	±	1.2	70.8	±	1.8
YFP-48aa-CFP *		10.5			70.0		
YFPα_1_/CFPβ_1_	NN	16.4	±	0.6	64.3	±	0.5
YFPα_2_/CFPβ_1_	NN	11.3	±	0.4	69.1	±	0.5
β_1_YFPα_1_CFP	Fusion	13.0	±	0.4	67.3	±	0.4
β_1_YFPα_2_CFP	Fusion	16.7	±	0.5	64.1	±	0.3
CFP/YFP		0.0	±	0.2	n/a		

Red marked values are FRET efficiencies of constructs were donor and acceptor were separated by helical linkers of 18, 28, and 48 amino acids * [17].

#### sGC α_2_/β_1_ isoform

In the α_2_/β_1_ enzyme isoform co-expression of carboxy-terminally (CC) tagged subunits resulted in fully active enzyme complexes that showed a FRET efficiency of 20.4%. Co-expression of amino-terminally (NN) tagged subunits showed basal activity and a FRET efficiency of 11.3%. The combination of carboxy-terminally tagged α_2_ subunit and amino-terminally tagged β_1_ subunit (CN) showed basal enzyme activity and a FRET efficiency of 17.4%. The combination of the amino-terminally tagged α_1_ subunit and carboxy-terminally tagged β_1_ subunit (NC) resulted in the formation of a fully active enzyme complex that showed a FRET efficiency of 10.1% (see Fig. 4, Table 1).

#### Comparison of both sGC isoforms

Comparing the enzyme isoforms the values for FRET efficiency of the different fluorescent-tagged combinations are similar, except for the amino-terminally tagged variants (NN). In the α_1_/β_1_ enzyme isoform the carboxy-terminally tagged variants (CC) and the amino-terminally tagged variant (NN) show similar FRET efficiencies, while in the α_2_/β_1_ enzyme isoform the FRET efficiency of the carboxy-terminally tagged variants (CC) are nearly two-fold higher than the amino-terminally tagged variant (NN). Furthermore, in both enzyme isoforms carboxy-terminally tagged α subunit co-expressed with amino-terminally tagged β_1_ subunit (CN) shows significantly higher FRET efficiency than the combination of amino-terminally tagged α subunit and carboxy-terminally tagged β_1_ subunit (NC). This indicates that the β_1_ amino-terminal domain is in closer proximity to the catalytic domain than the α amino-terminal domain (see Fig. 4, Table 1). Reference FRET efficiencies and corresponding calculated distances in Å are included in Table 1 [17].

### Fusion of fluorescent-tagged sGC subunits

The results from the FRET experiments show that the combinations of amino- and carboxy-terminally (CN, NC) fluorescent-tagged dimerizing partner subunits led to FRET between the fluorescent proteins.

This surprising finding led us to speculate that fusion of the carboxy-terminus of one subunit to the amino-terminus of the other subunit may result in an enzyme complex not so different in conformation from its native counterpart. Our results had shown that fusion at the amino-terminus of the β_1_ subunit leads to an enzyme showing no NO sensitivity and activation by Cinaciguat (see Fig. 2). Since fusion at the amino-terminus of the α subunit had no influence on the stimulation of the enzyme by NO and NO/YC-1 (see Fig. 1 D, E), it was the most promising subunit for amino-terminal fusion. In addition, the fluorescent proteins tagged to the α amino-terminus and β_1_ carboxy-terminus (NC) were in sufficient proximity for energy transfer. On the basis of these results we fused the β_1_ subunit to the amino-terminus of the α subunit via a fluorescent protein. This leads to a single fusion protein consisting of the sGC β_1_ subunit followed by a fluorescent protein and the sGC α subunit.

**Figure 5:**
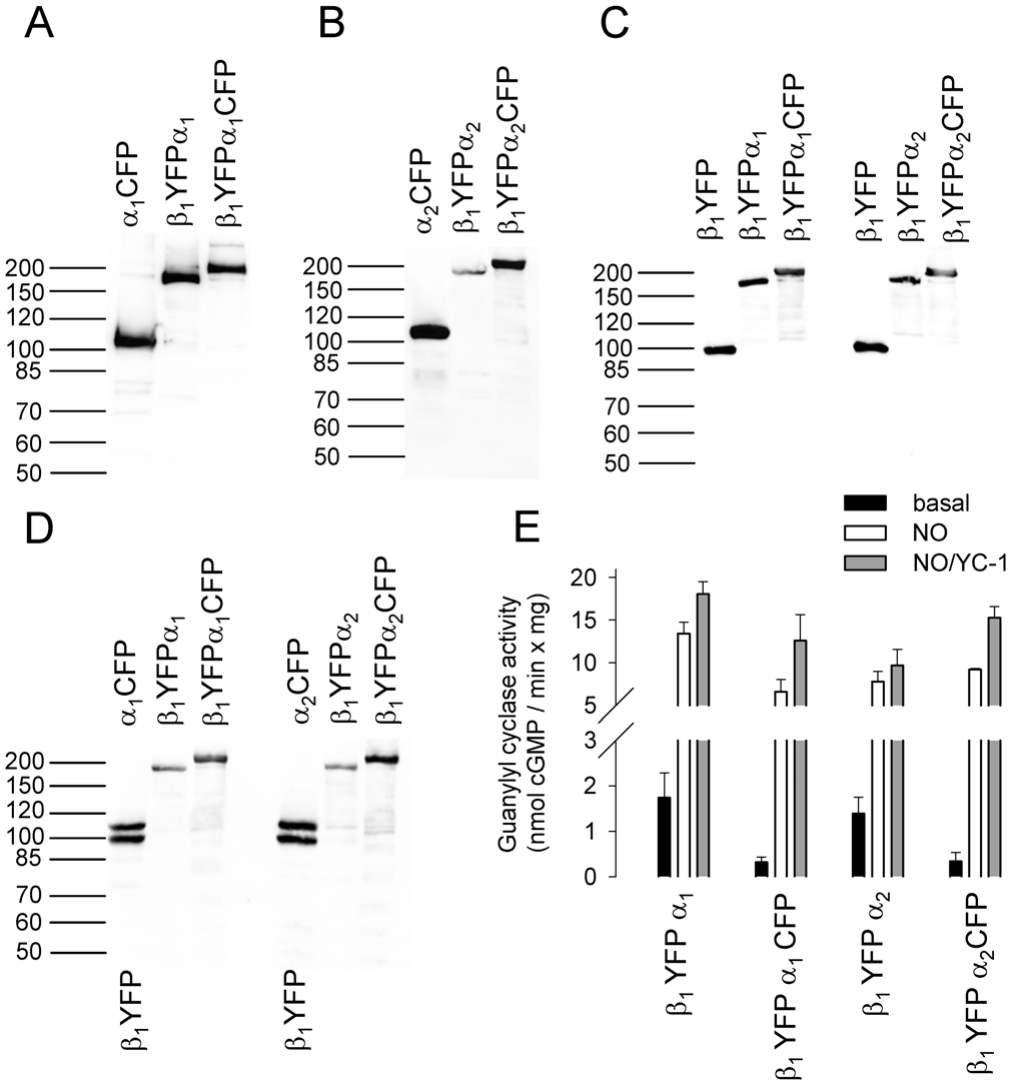
Analysis of fluorescent-conjoined sGC in cytosol of infected Sf9 cells. Western blot analysis with antibodies against the α_1_ subunit (**A**) α_2_ subunit (**B**) β_1_ subunit (**C**) and fluorescent protein (**D**). Specific sGC activity of the fluorescent-conjoined sGC variants (**E**). Data are mean ± S.E. of three independent experiments performed in duplicate.

After cloning and generation of recombinant baculovirus we expressed this monomeric construct in the Sf9/baculovirus system. Western immunoblots confirmed the expression of a ∼180 kDa protein, which corresponds to the expected molecular mass predicted from its amino acid sequence (Fig. 5 A–D). To our surprise, but in accordance with our predictions based on FRET experiments, enzyme activity assays revealed basal, as well as NO, and NO/YC-1 stimulated activity (Fig. 5 E). The construct was designated fluorescent-conjoined sGC.

To examine intramolecular FRET within one polypeptide chain we additionally fused the FRET donor CFP to the carboxy-terminus of the fluorescent-conjoined sGC, leading to an enzyme complex consisting of a β_1_ subunit followed by a fluorescent protein and an α subunit followed by another fluorescent protein with different spectral properties (see Fig. 5 A–D). This double fluorescent-conjoined sGC showed NO and NO/YC-1 stimulated activity as well (see Fig. 5 E).

In cytosolic fractions of Sf9 cells infected with one recombinant virus for the expression of fluorescent-conjoined sGC's, FRET was detected confirming that the fluorescent proteins at the α and β_1_ carboxy-terminus are in close proximity to each other. The measured FRET efficiencies are 13% and 16.7% for the α_1_ and α_2_ fluorescent-conjoined sGC isoform, respectively (see Fig. 4). These values are slightly lower than the FRET efficiencies of carboxy-terminally tagged heterodimeric isoforms (14.5% and 20.4%) (see Fig. 4 and Table 1), indicating a minor change in position or orientation of the fluorescent protein between the sGC subunits.

Enzyme activity assays showed that the fusion of the β_1_ carboxy-terminus to the α amino-terminus does not inhibit the formation of a functional enzyme complex and does not interfere with the activation mechanism of the enzyme. This result corroborates our prediction based on FRET that the amino-terminal domain of the α subunit is in proximity to the carboxy-terminal region of the β_1_ subunit.

### Dynamic changes on activation by NO

We were successful in generating fluorescent fusion proteins of individual subunits and fluorescent conjoined sGC's that dynamically reacted to activation by NO in enzyme activity determinations (see Fig. 1 and 5). Despite extensive testing we were not able to detect any changes in FRET efficiencies on addition of the NO-donor DEA/NO in concentrations that led to activation in enzyme activity determinations (data not shown). At the same time we were able to detect changes in FRET efficiency on addition of cGMP to YFP-GAFA-CFP which we cloned analogous to cGES-DE5 [16] (see Fig. 3).

## Discussion

Crystal structures of prokaryotic domains with high sequence similarities to functional domains of sGC have provided invaluable insights into the structure and possible functions of these isolated domains [5], [6], [8], [9], [13]. In the absence of structural information of the heterodimeric sGC holoprotein, the relative position of these domains to each other is unknown. In the current paper we use FRET analysis of fluorescent proteins fused to sGC subunits to study the overall organization of the enzyme as well as potential dynamic changes on activation by NO.

**Figure 6:**
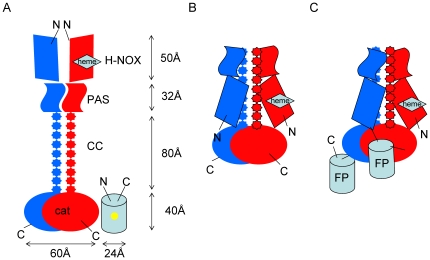
Proposed model of the heterodimeric sGC enzyme complex based on the results of FRET analysis and fusion of sGC subunits. The dimensions of the domains are given on the basis of the crystal structures of domain homologues (H-NOX, PDB ID 2O09 [9]; PAS, PDB ID 2P04 [13]; cat PDB ID 3ET6 [5]) and the structure of the coiled coil domain of the β_1_ subunit (PDB ID 3HLS [18]). Elongated model of the sGC (**A**). Model according to our results (**B**). Model of fluorescent-conjoined sGC (**C**). The β_1_ subunit is shown in red and the α subunit is shown in blue. cat - catalytic domain, CC - coiled coil region, PAS - Per-Arnt-Sim fold, HNOX - heme nitric oxide/oxygen binding domain.

Dimensions of sGC domains given in Figure 6 are determined on the basis of crystal structures of sGC homologues (H-NOX, PDB ID 2O09 [9]; PAS, PDB ID 2P04 [13]; cat, PDB ID 3ET6 [5]) and the structure of the coiled coil domain of the β_1_ subunit (PDB ID 3HLS [18]). The catalytic domains form a bulky structure at the carboxy-terminus of the subunits. FRET between fluorophores inside fluorescent proteins occurs at a distance of 10–80 Å [19]. The fluorescent proteins form a barrel that is 42 Å long and 24 Å in diameter [20]. The chromophores reside in the middle of the barrel leading to an actual distance between chromophores in wild-type GFP dimers of 25 Å [21]. Fusion of fluorescent proteins to the carboxy-termini of α and β subunits show FRET efficiencies of 15%–20% corresponding to a distance of 62 Å–66 Å (Table 1). These distances fit well to the distance between the carboxy-termini of crystallized catalytic domain dimers from *Chlamydomonas reinhardtii*, which are about 60 Å away from each other and oriented in opposite directions [5].

The coiled-coil domains consist of 65 amino acids forming an 80 Å long rigid structure [18]. On the amino-terminal side adjacent to the coiled-coil reside the PAS domains essential for dimerization followed by the H-NOX domains essential for enzyme stimulation by NO. On the basis of our FRET measurements the distance between the fluorophore attached to the β_1_ amino-terminus and the fluorophore attached to the α carboxy-terminus is in a range between 64–69 Å. This corresponds to a distance that a helical linker of 28 amino acids creates between two fluorescent proteins [17]. An elongated organization analogous to the membrane bound guanylyl cyclase where the regulatory amino-terminus is clearly separated from the catalytic domain is not compatible with our data. H-NOX domains, PAS-domains, coiled coil and catalytic region would result in a distance of over 200 Å (see Fig. 6 A). The distance from the start of the fluorescent protein to its fluorescent core is not more than 20 Å. To bring the fluorescent cores in a distance that is predicted by the calculated FRET efficiency 64–69 Å and that is below the 80 Å necessary for FRET to occur [19], the enzyme has to adopt a more compact conformation as shown in Figure 6 B.

Likewise the calculated distance based on our FRET results between a fluorophore attached to the α amino-terminus and a fluorophore fused to the β_1_ carboxy-terminus is in a range of 71–74 Å. This corresponds to a distance a helical linker of 48 amino acids creates between two fluorescent proteins [17]. Again this argues in favour of a compact enzyme structure where PAS and H-NOX domains of α and β subunits are folded back towards the catalytic domain thereby overcoming the distance created by the rigid coiled coil consisting of a helix of 65 amino acids [18].

Based on the ability of an amino-terminal construct of the β_1_ subunit (H-NOX) to inhibit activity of a heterodimer consisting only of the catalytic domains (α_cat_β_cat_), Winger and Marletta have proposed a direct interaction of the amino-terminal region of α_1_ with the catalytic domains [15]. Analysis of our FRET measurements supports the concept that sGC is structurally organized in a way that allows for direct interaction of the amino-terminal (H-NOX) domains of β_1_ with the carboxy-terminal catalytic region [15].

The function of the H-NOX domains of α_1_ and α_2_ subunits is not very clear. H-NOX-β_1_ is known to bind heme via a co-ordinating histidine [22], [23]. H-NOX-α lacks this histidine and heme since H-NOX-β_1_ constructs are sufficient for heme binding [24] and deletion of the H-NOX domain in α_1_ does not alter heme spectra of the purified heterodimeric enzyme [25]. Thus “heme binding” in the acronym H-NOX does not seem entirely correct for the H-NOX domain of α_1_ and α_2_. However, other members of the H-NOX superfamily also lack the heme co-ordinating histidine residue (e.g. in bacterial H-NOX versions from *Rhodobacter sphaeroides* and *Magnetococcus* species (Rhsp 22958463, Mcsp 22999020, [12]). It is conceivable that these domains play a conserved role in evolution and serve as binding sites for as yet unidentified substances rather than NO heme. Our FRET analysis and the enzymatically active fluorescent conjoined fusion protein suggest that the H-NOX domains of the α subunits could also have a direct influence on the catalytic domain. Because deletion of the H-NOX-α domains has surprisingly little influence on the holoenzyme [25], [26] a modulation of guanylyl cyclase activity in analogy to the H-NOX domain of β_1_ is conceivable in the presence of an as yet not identified ligand or binding protein.

Rothkegel *et al.* 2007 used an elegant bimolecular fluorescence complementation (BiFC) approach in a cGMP reporter cell line to study heterodimerization of sGC α_1_ and β_1_ subunit [27]. Fluorescence complementation and catalytic activity occurred when both halves of the fluorescent protein were fused to the respective *amino-terminus* of both sGC subunits. When both halves of the fluorescent protein were fused to the respective *carboxy-terminus* of both subunits, this led to fluorescence complementation but no catalytic activity. While the study was intended to study heterodimerization of sGC subunits, it is interesting to compare the results using BiFC with regard to the structural organization of sGC to our FRET approach. Both techniques concordantly indicate that the amino-terminal regions are closely interacting and that the carboxy-terminal regions are closely interacting in the α_1_/β_1_ enzyme isoform. Since steric constraints can prevent the association of the two halves of a fluorescent protein in BiFC, even when they are close to each other [28], the data by Rothkegel do not argue against our findings that the amino-terminal and carboxy-terminal parts of the various subunits are in close proximity.

To additionally test the proximity of the amino-terminus and carboxy-terminus suggested by our FRET results, we fused the carboxy-terminus of the β_1_ subunit to the amino-terminus of the α subunit using a fluorescent protein as linker that has an adjacent amino-terminal start and carboxy-terminal end (see Fig. 5). The resulting fluorescent-conjoined sGC enzyme showed specific sGC activity and stimulation by NO or YC-1 similar to the native sGC enzyme. Since fusion of sGC subunits led to an enzyme complex with the same properties as the native enzyme, these results further support the idea that the amino-termini and carboxy-termini of sGC are in close proximity to each other in the native enzyme. This is also supported by our recent finding that direct fusion of subunits of sGC leads to functional enzymes with preserved biochemical and pharmacological properties ([29] manuscript in preparation).

In contrast to Rothkegel [27], we were unable to demonstrate that the amino-terminal fusion of a fluorescent protein to the β_1_ subunit retained heme and thus NO-sensitivity. Other groups have also observed impaired heme binding with amino-terminal tags of the β_1_ subunit [30], [31]. With regard to catalytic enzyme activity it is noteworthy that fusion of the fluorescent moieties to the carboxy-terminus of α_1_ and β_1_ subunits suppressed cGMP formation in BiFC but not in our FRET study [27]. Since dimerization of the fluorescent halves in BiFC is demonstrated by fluorescence, we hypothesize that this additional dimerization interface provided by both fluorescent halves of the fluorescent protein induces a steric change e.g. closure of the substrate binding site.

Neither did Rothkegel report any experiments where the effect of NO was tested on BiFC induced fluorescence, nor did we observe changes in FRET efficiencies induced by addition of NO to the various constructs. Our current hypothesis is therefore that the conformational changes induced by NO are rather subtle. This could argue in favour of proposed activation mechanisms that are based on modifications of residues by nitrosylation in the catalytic region rather than extensive conformational changes [32]. NO binding to heme apparently does not change the relationship of the fluorophores fused carboxy-terminally to α and β subunits making a drastic conformational change in the catalytic region very unlikely. Interpretation of the data including the fluorophore attached to the β_1_ subunit is more complicated since this led to a loss of heme where NO binds (see Fig. 2). It would be conceivable that the H-NOX domain of the β_1_ subunit with a heme group has a distance to the catalytic domain that changes on activation by NO. This would be the case in a model where NO leads to derepression of the β_1_ H-NOX domain. We are currently trying to use heme as a FRET acceptor and a blue fluorescent protein as a FRET donor in fluorescence lifetime measurements (FLIM-FRET) to be able to circumvent fusion of a fluorescent protein to the amino-terminus of the β_1_ subunit. While the data in the current manuscript provide an ideal starting point for such a strategy, our first series of experiments indicate that this is a demanding complex project because heme in sGC may not be an ideal FRET acceptor. Among other problems NO binding changes absorbance properties of heme and heme containing subunits and apoforms of the β_1_ subunit co-exist *in vivo*.

Fluorescent-conjoined sGCs represent to our knowledge the first example where a fluorescent protein links two different proteins to yield a functionally active enzyme. In addition, to having served as a test system for analyzing the overall structural organization of sGC the fluorescent-conjoined sGC consisting of one protein chain containing the β_1_ and α subunits in series may serve as a powerful tool for research in the NO/cGMP field.

## Materials and Methods

### Materials

3-(5-Hydroxymethyl-2-furyl)-1-benzylindazole (YC-1) and 2,2-Diethyl-1-nitroso-oxyhydrazine (DEA/NO) and all other chemicals, were obtained from Sigma in the highest grade of purity. Products for Sf9 cell culture were from Invitrogen. Protein purification was done using ÄKTA Purifier FPLC-System (GE Healthcare). *Strep*-Tactin Superflow was purchased from IBA-Biotagnology.

### Cloning of Fluorescent Tagged Guanylyl Cyclase Subunits and Generation of Recombinant Baculovirus

Recombinant baculoviruses of respective fluorescent tagged proteins were generated according to the BAC-TO-BAC™ System (Invitrogen). Further information concerning the cloning of fluorescent tagged proteins can be found in the supplement.

### Sf9 Cell Culture, Expression of Recombinant Guanylyl Cyclase Subunits and Preparation of Cytosolic Fractions

Sf9 cells (Invitrogen Cat.No. 11496-015) were cultured in Sf-900 II serum-free medium supplemented with 1% penicillin/streptomycin and 10% fetal calf serum. Spinner cultures were grown at 27°C at 140 rpm shaking and diluted to 2×10^6^ cells/ml for infection. 20 ml of cell solution were infected with the respective recombinant baculovirus stock. After 74 h cells were harvested and collected by centrifugation (4000×*g* for 10 min at 4°C). All following steps were performed at 4°C or on ice. The cell pellet was resuspended in 4 ml of homogenization buffer containing 50 mM TEA/HCl, pH 7.6, 1 mM EDTA, pH 8.0, 10 mM dithiothreitol, and complete™ protease inhibitor cocktail (Roche). The cells were lysed by sonication. Cytosolic fractions were obtained by centrifugation for 30 min at 21,000×*g* at 4°C.

### Determination of Protein Concentration, Guanylyl Cyclase Activity, SDS-PAGE, and Western Immunoblotting

Protein concentrations were determined by the method of Bradford using bovine serum albumin as standard. sGC activity of Sf9 cytosol (approximately 60 µg of protein per assay tube) was determined using isotope labelled [^32^P]GTP as described previously in [25] by incubation for 10 min at 37°C in the presence of 1 mM cGMP, 0.5 mM [^32^P]GTP (about 0.2 µCi), 3 mM MgCl_2_, 50 mM TEA/HCl, pH 7.4, 0.25 g/liter creatine kinase, 5 mM creatine phosphate, and 1 mM 3-isobutyl-1-methylxanthine in a total volume of 0.1 ml. Reactions were started by the addition of protein and incubation at 37°C. All experiments were stopped by ZnCO_3_ precipitation, and purification of the enzyme-formed cGMP was performed as described previously [33]. Basal enzyme activity measurements were performed in the absence of NO or YC-1. NO stimulated measurements were performed in the presence of the NO donor DEA/NO, and NO/YC-1-stimulated enzyme activity measurements were performed in the presence of both DEA/NO and YC-1. YC-1 was dissolved in 25% (v/v) DMSO so that the final DMSO concentration in the enzyme assay did not exceed 2.5% (v/v). At this concentration no effects of DMSO on enzyme activity were observed. DEA/NO was dissolved in 10 mM NaOH, which also did not affect the enzyme activity.

For immunoblotting, protein fractions were subjected to 10% SDS-PAGE and then transferred electrophoretically to a nitrocellulose membrane. The membrane was reversibly stained with Ponceau S, and unspecific binding sites were saturated by immersing the membrane for 1 h in TBST buffer (10 mM Tris/HCl, pH 8.0, 150 mM NaCl, 0.05% Tween 20) containing 5% nonfat dry milk. The membranes were incubated for 1 h in TBST buffer containing antibodies directed against the β_1_, α_1_ (Sigma) and α_2_ [34] subunit of soluble guanylyl cyclase. Fluorescent protein tagged sGC subunits were also detected by antibodies against GFP (Clontech). The membranes were washed three times for 10 min with TBST and subsequently incubated for 1 h with horseradish peroxidase-labeled anti-rabbit IgG antibodies (Cell Signaling). After three washes with TBST the membranes were processed with the ECL Western blotting detection system according to the recommendations of the manufacturer (Roche).

### Protein Purification

All purification steps were performed at 4°C. The cell pellet from 500 ml of cell solution infected with the respective recombinant baculovirus was homogenized by sonification in 30 ml homogenization buffer containing 50 mM TEA/HCl, pH 7.4, 10 mM DTT, 1 mM EDTA and complete™ protease inhibitor cocktail (Roche). Avidin (250 nM, IBA) was added to the homogenate and incubated for 30 min at for 4°C. The homogenate was centrifuged at 15,000 g for 2 h and 30 ml of the supernatant was collected. All chromatographic steps were performed on an ÄKTA purifier system (GE Healthcare). The supernatant was immediately applied to a Strep-Tactin® Superflow® high capacity (4 ml volume, IBA) column (C 10/10 and adapter AC 10, GE Healthcare) at 1 ml/min. The Strep purification was done using the Strep-tag® Protein Purification Buffer Set and according to manufactures protocol (IBA). Fractions showing absorption at 280 nm were pooled and applied immediately with a flow rate of 15 ml/min to a HiPrep 26/10 Desalting column (GE Healthcare) that was equilibrated 5 CV with IEX-1 (50 mM TEA, 10 mM DTT, 1 mM Benzamidin, pH 8.0) buffer before use. Elution was carried out with 2 CV at 15 ml/min with IEX-1 buffer and fractionated in 1.0 ml. Fractions showing absorption at 280 nm were pooled and applied immediately with a flow rat of 2 ml/min to a Mono Q 5/50 GL column (GE Hethcare) that was equilibrated 10 CV with IEX-1 buffer before use. The column was washed (10 CV) at 2 ml/min with IEX-1. Elution was carried out with 10 CV at 2 ml/min with a linear gradient running from zero to 100% IEX-2 buffer (50 mM TEA, 10 mM DTT, 1 mM Benzamidin, 1 M NaCl, pH 8.0) and fractionated in 0.5 ml. Peak fractions showing absorption at 280 nm were pooled and concentrated using Amicon centrifugal filter units (30,000 MW). Purified sGC was detected in pooled fractions using sensitive coomaissie blue staining, analysis of absorption spectra were done on a UV-VIS Spectrophotometer (Cary 50).

### Spectrofluorometer Study of FRET

The spectrofluorometric study was carried out on a Varian spectrofluorometer. Cytosolic fractions from Sf9 cells infected with the respective recombinant baculovirus were investigated at 37°C using a heated cuvette mount. For spectral analysis the spectrofluorometer was set at the scan mode of 1 nm wavelength intervals, emission PMT voltage 600 V, the emission slit width and excitation slit width 5 nm and 5 nm, respectively. For measurement of FRET by sensitized emission using three channels the spectrofluorometer was set at the simple reads mode of 0.2 sec integration time with an emission PMT voltage of 600 V, the emission slit width and excitation slit width 5 nm and 5 nm, respectively.

### FRET Measurement and Calculation

FRET measurements were done using the sensitized emission method with three channels. In the donor channel the excitation wavelength of CFP was selected at 436 nm on the blue site of the CFP excitation spectrum to avoid excessive cross excitation of the acceptor YFP. The emission of CFP was detected at 476 nm. In the acceptor channel the excitation wavelength of YFP was 515 nm and the emission wavelength 527 nm. Therefore, the FRET channel was selected at 436 nm excitation and 527 nm emission (see supplementary Fig. S1). Before the determination of FRET fluorescent samples were diluted to equal fluorescence intensity in the YFP channel with homogenization buffer. In order to determine the amount of background fluorescence due to excitation of cytosolic components, the protein content of the diluted samples was determined and the respective background fluorescence of cytosolic fractions of uninfected Sf9 cells was subtracted. Using this background subtraction the values for CFP fluorochrome in the YFP channel and YFP fluorochrome in the CFP channel were effectively zero. Corrected FRET (FRET^C^) was calculated using the equation: 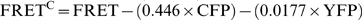 (see reference[35]), where FRET, CFP, and YFP correspond to background subtracted fluorescence intensities of cytosolic fractions co-expressing CFP and YFP acquired through the FRET, CFP, and YFP channels, respectively. 0.446 and 0.0177 are the fractions that were calculated for the bleed-through of CFP and YFP, respectively, through the FRET channel. These values of bleed-through were similar to those described by other researches for the CFP/YFP pair [36], [37]. The negative FRET^C^ values obtained in control experiments are due to slight overestimation of the bleed-through coefficients. The corrected FRET values obtained above can be affected by variations in CFP and YFP fluorescence intensities due to the expression level of CFP and YFP in different samples. To compare FRET values between different samples, the FRET efficiency (apparent Efficiency, E%) was calculated according to the equation: 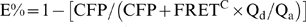 (see reference [38], [39]) where FRET^C^ is the corrected FRET from the equation above and CFP corresponds to the background subtracted fluorescence intensities of cytosolic fractions co-expressing CFP and YFP acquired through the CFP channel, Q_d_ and Q_a_ are the donor and acceptor quantum yields, respectively ( Q_d_ = 0.4, Q_a_ = 0.61).

The distance (r) between donor and acceptor molecule is calculated using the equation:
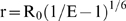
where R_0_ is the Förster radius at which the energy transfer from donor to acceptor is 50% (R_0 CFP/YFP_ = 4,9 nm). The Förster radii were calculated elsewhere [40] according to Förster [41] under the assumption of complete dynamic isotropic orientational averaging of donor and acceptor using a κ^2^ value of ^2^/_3_.

## Supporting Information

Figure S1 Normalized fluorescence excitation and emission spectra of fluorescent proteins and fluorescent tagged sGC subunits. Excitation spectra of ECFP (cyan dashed line) and emission spectra of ECFP (cyan solid line), excitation spectra of EYFP (yellow dashed line) and emission spectra of EYFP (yellow solid line) are preserved after fusion to sGC subunits. Spectral properties of the respective fluorescent tagged sGC subunits are shown with black lines. Fluorescence emission spectrum of the sGC α_1_YFP/β_1_CFP heterodimer (red line) after excitation at 436 nm. Shaded bars indicate the wavelength for excitation and emission used in sensitized emission FRET study.
(1.18 MB TIF)

Methods S1 Cloning of fluorescent tagged proteins and generation of Recombinant Baculovirus.
(0.04 MB DOC)
