# Supplementary figures and images for: Fluorescent Fusion Proteins of Soluble Guanylyl Cyclase Indicate Proximity of the Heme Nitric Oxide Domain and Catalytic Domain
Source: PLoS One. 2010 Jul 15;5(7):e11617. doi: 10.1371/journal.pone.0011617 (PMC2904703; doi:10.1371/journal.pone.0011617)

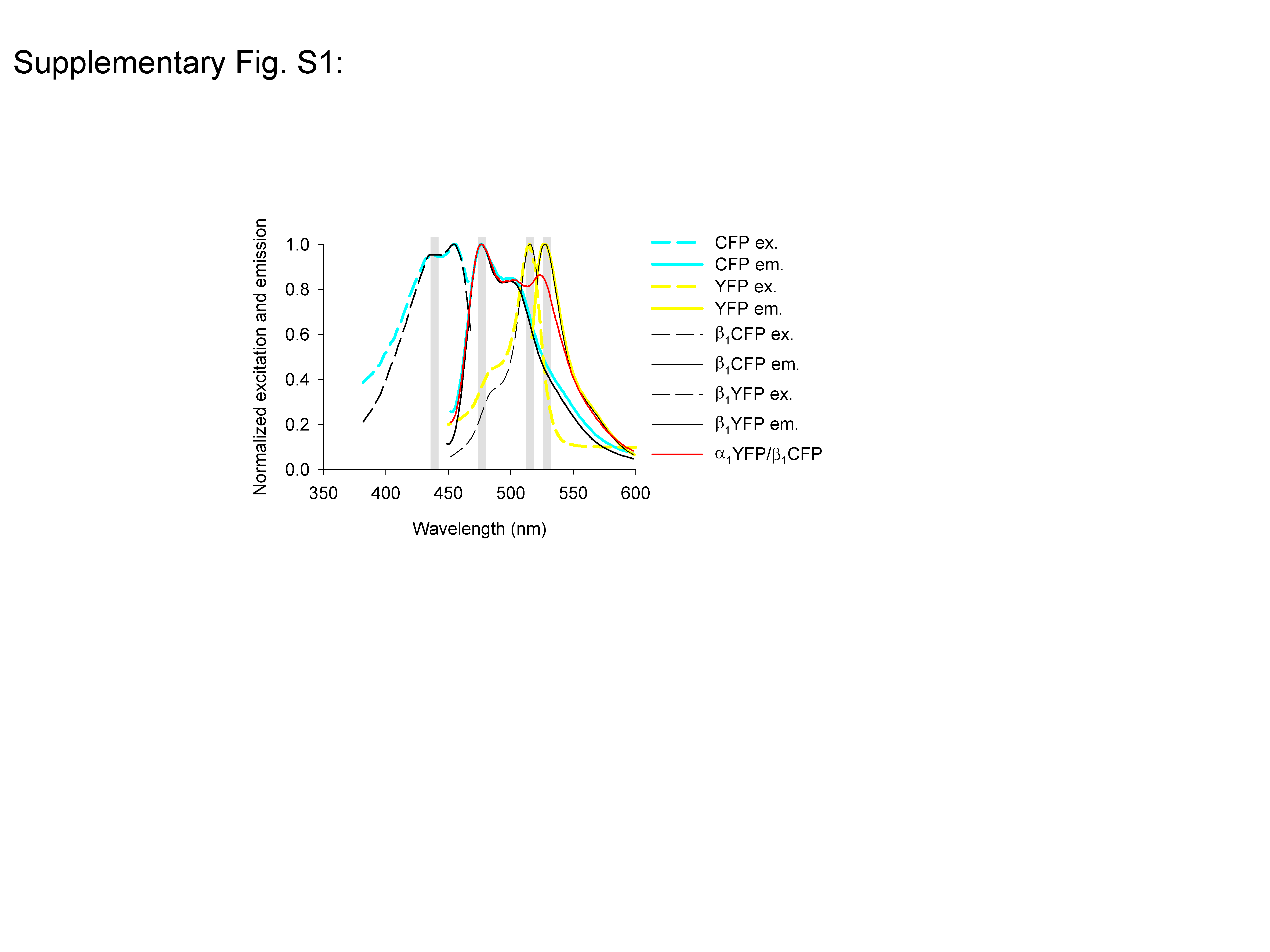

Supplement: Figure S1 — Normalized fluorescence excitation and emission spectra of fluorescent proteins and fluorescent tagged sGC subunits. Excitation spectra of ECFP (cyan dashed line) and emission spectra of ECFP (cyan solid line), excitation spectra of EYFP (yellow dashed line) and emission spectra of EYFP (yellow solid line) are preserved after fusion to sGC subunits. Spectral properties of the respective fluorescent tagged sGC subunits are shown with black lines. Fluorescence emission spectrum of the sGC α1YFP/β1CFP heterodimer (red line) after excitation at 436 nm. Shaded bars indicate the wavelength for excitation and emission used in sensitized emission FRET study. (1.18 MB TIF) [file pone.0011617.s001.tif]
