# Supplementary material for: Fluorescent Fusion Proteins of Soluble Guanylyl Cyclase Indicate Proximity of the Heme Nitric Oxide Domain and Catalytic Domain
Source: PLoS One. 2010 Jul 15;5(7):e11617. doi: 10.1371/journal.pone.0011617 (PMC2904703; doi:10.1371/journal.pone.0011617)
Supplement: Methods S1 — Cloning of fluorescent tagged proteins and generation of Recombinant Baculovirus. (0.04 MB DOC) [file pone.0011617.s002.doc]

**Methods S1**

***Cloning of fluorescent tagged proteins and generation of Recombinant Baculovirus-*** Recombinant baculoviruses of respective fluorescent tagged proteins were generated according to the BAC-TO-BAC^TM^ System (Invitrogen).

*sGC α_1_ fluorescent proteins*

The sGC α_1_ subunit was amplified from rat cDNA using the primer pair: P178 (5’-ACA CCG GCT AAT AAG GAG GAA ACC AC-3’) and P179 (5’-ATC TAC CCC TGA GGC CTT GCC TAA GAA-3’). The resulting PCR product was subcloned into pcDNA3.1/V5/His-TOPO. From this construct the α_1_ cDNA was cloned HindIII / StuI into HindIII / SmaI pEGFP‑N1 and HindIII / XhoI into HindIII / SalI pECFP-N1. SGC α_1_ CFP was cloned NheI / XbaI into SpeI / XbaI pFASTBAC. In pFASTBAC CFP was exchanged with YFP from pEYFP-N1 using the restriction sites AgeI / XbaI. The predicted molecular mass of YFP α_1_ is 104 kDa.

For cloning of CFP α_1_, rat α_1_ subunit in pcDNA3.1/V5/His-TOPO was brought BsrGI / XbaI into pECFP‑C1. CFP α_1_ was cloned XbaI / NheI into XbaI of pFASTBAC. In pFASTBAC ECFP was exchanged with EYFP using BsrGI restriction site in a nondirectional cloning strategy with subsequent orientation test. The predicted molecular mass of α_1_ YFP is 107 kDa.

*sGC α_2_ fluorescent proteins*

SGC α_2_ subunit was cloned BamHI / SspI from pFASTBAC [41] into BglII / BamHI (Klenow fill in) in pEYFP-N1. SGC α_2_ subunit from α_2_ in pEGFP-N1 was cloned Eco47III / NotI into pECFP-N1 to get the α_2_ CFP construct. α_2_ CFP was cloned Eco47III / NotI into StuI / NotI pFASTBAC vector. ECFP was exchanged with EYFP from pEYFP‑C1 using AgeI / KpnI restriction sites. The predicted molecular mass of α_2_ YFP is 108 kDa.

SGC α_2_ rat was cloned BstEI (Klenow fill in) / BamHI from pFASTBAC [41] into pEYFP-C1 EcoRI (Klenow fill in) / BamHI. YFP α_2_ construct was cloned AgeI / SpeI into AgeI / XbaI of pFASTBAC vector. EYFP was exchanged in pFASTBAC with ECFP from CFP β_1_ construct using AgeI / BstI restriction sites. The predicted molecular mass of YFP α_2_ is 113 kDa.

*sGC β_1_ fluorescent proteins*

The β_1_ subunit cDNA was amplified by RT-PCR from rat heart using Clontech cDNA Polymerase Mix and the primer pair P141 (5’-CCG ACA CCA TGT ACG GTT TTG TGA-3’) and P180 (5’-GGG CCC AGT TTT CAT CCT GGT TTG TTT CCT-3’). The resulting PCR product was subcloned into pcDNA3.1/V5/His-TOPO. From this construct the β_1_ subunit cDNA was cloned HindIII / ApaI into pEGFP-N1 and also pEYFP-N1. The predicted molecular mass of β_1_ CFP is 102 kDa.

β_1_ YFP construct was cloned SpeI / XbaI from pEYFP-N1 into pFASTBAC. EYFP was exchanged AgeI / XbaI with ECFP from pECFP-N1. β_1_ YFP was cloned SalI/XbaI into pECFP-N1 to get a CFP β_1_ YFP construct. From CFP β_1_ YFP the YFP gene was deleted SmaI / NotI and religated to get a CFP β_1_ construct. ECFP in CFP β_1_ was exchanged NheI / EcoRI with EYFP from pEYFP-C1. CFP β_1_ and YFP β_1_ were cloned NheI / XbaI into SpeI / XbaI of pFASTBAC vector. The predicted molecular mass of CFP β_1_ is 101 kDa.

*YFP-GAFA-CFP*

CDNA coding for the GAF A domain was amplified from human placenta cDNA (clontech) using the primer pair (P254 5’- CGT ACG AGG AAT TAG TGA AGG ATA TTT CTA -3’) and P253 (5’- CAA TAC CAC AAA ATG CCA AAT AAG -3’) and subcloned into pCR 4 TOPO. From pCR4-TOPO a 500 bp fragment was cut AgeI / EcoRI and cloned into pECFP‑N1 vector. GAF A CFP was cloned BsiWI / NotI into BsrGI / NotI pEYFP-N1. The cDNA coding for YFP-GAFA-CFP was cloned EcoRI / NotI into pFASTBAC vector.

*Fluorescent-conjoined sGC constructs*

The rat sGC α_1_ subunit was amplified from pcDNA3.1/V5/His-TOPO using primer designed with internal restriction sites. The primer pair used were: P270 (BsrGI_a1_for) (5’- GAT CGT GTA CAA GAT GTT CTG CAG GAA GTT CAA -3’) and P271 (Xba_a1_rev) (5’- GAT CGT CTA GAA TCT ACC CCT GAG GCC TTG CC-3’), and P269 (Xba_a1_Stop_rev) (5’- GAT CGT CTA GAT TAA TCT ACC CCT GAG GCC TTG CC-3’), respectively. The resulting PCR product was subcloned into pCR2.1 TOPO. From this construct the α_1_ cDNA was cloned BsrGI / XbaI into β1 YFP in pEYFP-N1 to get a β_1_YFPα_1_ construct with stop codon and a β_1_YFPα_1_ construct without stop codon for further cloning of the ECFP gene 3’ of the α_1_ subunit cDNA. The fluorescent-conjoined sGC β_1_YFPα_1_ without stop codon was cloned NheI / XbaI into pECFP-N1. Since ECFP was not in frame the construct was cut SacII / SalI and Mung bean treated to get blunt ends for religation leading to an in frame ECFP gene. Both constructs β_1_YFPα_1_ and β_1_YFPα_1_CFP were cloned SpeI / XbaI into pFASTBAC vector. The predicted molecular masses of β_1_YFPα_1_ and β_1_YFPα_1_CFP are 176 kDa and 205 kDa, respectively.

α_2_ cDNA in pEYFP-C1 (see above) was linearized with BsrGI overnight and partially digested with NheI to delete an 731 bp region containing EYFP from the vector construct with the α_2_ cDNA (6334 bp). In this construct we ligated a 2684 bp fragment from NheI / BsrGI digest β_1_ rat in pEYFP-N1 (see above) resulting in the fluorescent-conjoined β_1_YFPα_2_ (in pEYFP-C1).

α_2_ in pEYFP-C1 (see above) was cut NheI / SspI. The resulting 297 bp fragment containing part of the α_2_ cDNA right before the endogenous stop codon was cloned in frame with ECFP into the pECFP-N1 vector cut NheI / BamHI (Klenow fill in). This construct (designated “pECFP-N1 SspI”) was cut NheI and a 4692 bp resulting from NheI digest of the β_1_YFPα_2_ construct (see above) was inserted. The resulting fluorescent-conjoined sGC construct was designated β_1_YFPα_2_CFP (in pEYFP-C1).

The β_1_YFPα_2_CFP (see above) was cloned SpeI / NotI into pFASTBAC. This construct was used for generation of recombinant baculoviruses. In addition the construct was cut NheI and ligated with a 400 bp fragment from NheI / SpeI digested β_1_YFPα_2_ in pEYFP-C1 containingthe natural α_2_ stop codon (see above). The predicted molecular masses of β_1_YFPα_2_ and β_1_YFPα_2_CFP are 185 kDa and 211 kDa, respectively.
